# Transcultural adaptation and psychometric properties of the spanish version of the therapeutic relationship Assessment Scale-Nurse

**DOI:** 10.1186/s12912-023-01412-8

**Published:** 2023-07-28

**Authors:** Juan Roldán Merino, Joana Coelho, Francisco Sampaio, Zaida Agüera, Montserrat Puig Llobet, Teresa Lluch Canut, Oscar Rodriguez Nogueira, Ana Ventosa Ruiz, Carlos Sequeira, Antonio R. Moreno Poyato

**Affiliations:** 1 Campus Docent Sant Joan de Déu – Fundació Privada, School of Nursing, C/ Sant Benito Menni, 18-20, Sant Boi de Llobregat (Barcelona), 08830 Spain; 2 Universitat de Barcelona, Mental Health, Psychosocial and Complex Nursing Care Research Group (NURSEARCH), Gran Via de les Corts Catalanes, 585, Barcelona, 08007 Spain; 3 Portuguese Red Cross Northern Health School, Rua da Cruz Vermelha Cidacos-Apartado 1002, Oliveira de Azeméis, 3720-126 Portugal; 4 CINTESIS@RISE, Nursing School of Porto (ESEP), Rua Dr. Plácido da Costa, Porto, 4200-450 Portugal; 5 Nursing School of Porto, Rua Dr. António Bernardino de Almeida, 830, 844, 856, Porto, 4200-072 Portugal; 6 Universitat de Barcelona, Facultat de Medicina i Ciències de la Salut, Departament d’Infermeria de Salut Pública, Salut Mental i Salut Materno-Infantil, Gran Via de les Corts Catalanes, 585, Barcelona, 08007 Spain; 7 Bellvitge Biomedical Research Institute (IDIBELL), Neurosciences Programme, Psychoneurobiology of Eating and Addictive Behaviors Group, Gran Via de l’Hospitalet, 199, Hospitalet de Llobregat (Barcelona), 08908 Spain; 8 Instituto de Salud Carlos III, CIBER Fisiopatología de la Obesidad y Nutrición (CIBERObn), C/ Sinesio Delgado, 4, Madrid, 28029 Spain; 9 Universidad de Léon, Campus de Ponferrada, Department of Nursing and Physiotherapy, Av. de Astorga 15, Ponferrada (León), 24400 Spain; 10 Universidad de León, SALBIS Research Group, Av. de Astorga, Ponferrada (León), 24400 Spain; 11 Benito Menni Complex Assistencial en Salut Mental, Sant Boi de Llobregat, C/ Dr. Antoni Pujadas, 38, Sant Boi de Llobregat (Barcelona), 08830 Spain

**Keywords:** Mental Health nursing, Psychometrics, Nurse-patient relations

## Abstract

**Background:**

The nurse-patient therapeutic relationship is considered a pillar of mental health nursing, contributing to improved person-centered care and shared decision making with the patient. Given the importance of the nurse-patient therapeutic relationship, appropriate evaluation instruments are required to assess its quality. The aim of this study was to adapt and validate the Spanish version of the Therapeutic Relationship Assessment Scale-Nurse.

**Methods:**

A translation and back-translation of the scale was carried out. To analyze the psychometric properties, the scale was administered to 213 nurses working in the field of mental health care. Temporal stability or test-retest was examined by means of the intraclass correlation coefficient (ICC) in a sample of 100 nurses.

**Results:**

Confirmatory Factor Analysis revealed a four-factor structure identical to the original version, with some poor model fit indices. The ordinal alpha values for the total scale and the four factors were 0.939, 0.654, 0.798, 0.801, and 0.866, respectively. The intraclass correlation coefficient was 0.928 (95% CI: 0.893–0.952).

**Conclusions:**

The results show that the Spanish version of the Therapeutic Relationship Assessment Scale-Nurse is reliable for determining the quality of the therapeutic relationship that mental health nurses can establish with their patients. However, more studies are needed to analyse the model fit of the instrument’s factor structure in the Spanish population.

**Supplementary Information:**

The online version contains supplementary material available at 10.1186/s12912-023-01412-8.

## Background

The nurse-patient therapeutic relationship (TR) is considered a mainstay in mental health nursing [1], contributing to better person-centered care and shared decision making with the patient [2]. The TR constitutes a vehicle through which nurses carry out their interventions in clinical practice and improve the health of people with mental health problems [3].

From a theoretical perspective, in the 1950s, Peplau formulated a nursing theory named Interpersonal Relations in Nursing [4] For Peplau, nursing practice is based on an interpersonal process between the nurse and the patient, through which the nurse must identify the patient’s needs, thus promoting personal growth and producing changes that have a positive influence on the patient’s life [5].

Undoubtedly, multiple factors affect and condition the establishment of a TR in the clinical practice of mental health nurses [6]. Contextual factors may include the structure and environment where the relationship takes place, the patient’s condition, the dynamics of the service, and its rules and regulations [7]. In addition, professional factors specific to the nurse are in play, related to their general competence for evidence-based practice [8] or more specific aspects such as their ability to create a conducive environment, use an appropriate verbal approach, offer help, work together with the patient and, of course, display an open attitude toward the relationship [7]. Within these nursing factors, the literature points to understanding, interest, availability, individuality, authenticity, warmth, respect, and self-knowledge as the basic pillars for the nurse to effectively build the relationship [9, 10].

Given the clinical relevance of the TR for mental health nursing, it is necessary to have instruments to assess its quality. Several evaluation instruments are described in the empirical literature, most of which are constructed using a psychodynamic model approach, such as the Working Alliance Inventory [11], the Helping Alliance Questionnaire [12], and the California Psychotherapy Alliance Scale [13]. Other instruments with a more eclectic approach have been validated for more specific settings. The Therapeutic Engagement Questionnaire [14] was developed for use in acute inpatient mental healthcare settings and focused on nurse-patient interactions in the overall environment and atmosphere of the ward. The Scale to Assess the Therapeutic Relationship [15] was constructed for the measurement of therapeutic relationships in a community setting. The most widely used instrument to measure therapeutic relationships is the Working Alliance Inventory Short [11]. The WAI-S has been translated into over 15 languages [16] and has the most data on its reliability in different populations [17]. However, given its construction centered on the therapeutic alliance as an element of psychotherapy improvement [18], it does not accurately fit the context of mental health nursing practice [14, 19].

Also, a new instrument has been published in Portugal called the Therapeutic Relationship Assessment Scale-Nurse (TRAS-Nurse) [19], which has been constructed for nursing practice and is specifically aimed at assessing the quality of the nurse-patient therapeutic relationship in different mental health care settings and beyond the psychotherapeutic concept followed by most of the existing instruments in the literature. TRAS-Nurse obtained adequate validity and reliability for the 25-item instrument distributed by four factors: empathy (five items), self-knowledge (six items), involvement (eight items), and orientation (six items). Furthermore, TRAS-Nurse can also be used as a one-factor structured instrument with 25 items [19].

Considering the lack of instruments that assess the quality of the TR, specifically from and for mental health nursing and for different mental health care settings, it is considered that the TRAS-Nurse tool meets these conditions in its Portuguese version and that, therefore, it can be of great use for application in other international contexts.

## Methods

### Aim

This study aimed to translate, culturally adapt, and evaluate the psychometric properties of the Spanish version of the Therapeutic Relationship Assessment Scale-Nurse (TRAS-Nurse) [19].

### Design

A cross-sectional descriptive psychometric study was carried out in two phases. In the first phase, the TRAS-Nurse scale was translated and adapted to Spanish and, in the second phase, the metric properties of the Spanish version of the TRAS-Nurse scale were analyzed.

### Participants and study setting (sample size)

The participating nurses were recruited through the Spanish Association of Mental Health Nursing and the Catalan Association of Mental Health Nursing using a convenience sampling method. The sample consisted of 213 nurses who met the following inclusion criteria: nurses currently working in the mental health field and with at least one year’s experience in any mental health service (hospital, community).

Data collection was carried out from February to July 2022. An on-line form was designed through the REDCap platform, consisting of three sections. The first section contained the information sheet on the study and the informed consent form. If the participant consented and signed, the form led to the second section. The second section collected sociodemographic variables and the third section corresponded to the Spanish version of the “Therapeutic Relationship Assessment Scale-Nurse” and the Working Alliance Inventory Short therapist version [11].

The sample size was determined based on current recommendations that consider the minimum number of subjects for CFA to be 200 [20, 21]. Moreover, it was estimated that a minimum of 80 participants would be needed to detect an intraclass correlation coefficient (ICC) of about 0.70 between the two administrations, assuming a confidence level of 95% and a power of 80% in a bilateral comparison [22].

### Variables and sources of information

Sociodemographic variables were age (continuous variable) and sex (categorical variable: women/men). As professional variables we collected mental health nursing specialty (categorical variable: yes/no), area in which the care activity was carried out (categorical variable: community level/hospital environment/undefined), work shift (categorical variable: morning/morning and afternoon/afternoon/night/rotating schedule), workday (categorical variable: complete/partial), type of contract (categorical variable: permanent/interim/replacement), years working in mental health (continuous variable), years worked in the current position (continuous variable), higher level university education (categorical variable: PhD/MSc/degree), and other specific mental health training (categorical variable: postgraduate/masters/none).

The evaluation instruments used were TRAS-Nurse scale and Working Alliance Inventory Short scale [11]:

- The original version of the TRAS-Nurse scale. It is conformed of 25 items each and is rated using a Likert-type scale ranging from 1 to 5 (1 - never, 2 - rarely, 3 - sometimes, 4 - often, 5 - always). The overall internal consistency of the scale showed adequate results (α = 0.93) [19]. This scale is composed of four factors: F1- Empathy, with five items, which represents the person’s acceptance and understanding (α = 0.86). F2-Self-konwledge, six 6 items, centered on reflection on the thoughts, feelings and behavior of nurses and the extent to which they can contribute to the therapeutic relationship (α = 0.85). F3-Involvement, with eight items, is focused on defining the person’s needs and expectations (α = 0.88). Finally, F4-Orientation, with six items, represents the definition of the roles of the nurse and the person in the therapeutic relationship (α = 0.78). Factor analysis revealed a unidimensional or four-factor structure [19].
- The Working Alliance Inventory Short scale (WAIS-S), therapist version [11]. This scale contains 12 items, each of which is evaluated by the health professional on a scale ranging from 1 (never) to 7 (always). The scale consists of three subscales of four items each: (a) bonding, (b) goals, and (c) tasks or activities. The higher the score, the stronger the therapeutic relationship. The Spanish version of the WAI-S has shown good reliability and validity, with Cronbach’s alpha values of 0.85 for the bond subscale, 0.81 for the goals subscale, 0.90 for the tasks’ subscale and 0.93 for the total scale [23].

### Procedure

The translation and back-translation processes were carried out according to the Standards for Educational and Psychological Testing [24]. First, authorization was requested from the author of the original scale for its adaptation to the Spanish population. Subsequently, the scale was translated from Portuguese into Spanish by two bilingual nurses whose mother tongue is Spanish and who were unfamiliar with the scale and the objectives of the study. A committee of seven experts, including nurses working in community and hospital settings, was created to review the semantic equivalence of these two versions and the first Spanish version of the scale was agreed upon. Thereafter, the Spanish version was back-translated into the original language by two translators whose mother tongue is Portuguese, in order to confirm its concordance with the original Portuguese version. Next, the original authors of the TRAS-Nurse scale examined the back-translation and compared it with the original version, finding no discrepancies that required modifications. Finally, a pilot test involving 15 nurses was conducted to assess the clarity and comprehension of the items, as well as the time required to complete the scale. After debriefing, it was not necessary to introduce changes in either format or content. Additional file 1 shows the semantic equivalence of the items from Portuguese to Spanish.

### Statistical analysis

The statistical program SPSS Statistics version 28 was used for data analysis, EQS version 6.3 was used for confirmatory factor analysis (CFA) [25].

### Construct validity

To analyze construct validity, a confirmatory factor analysis (CFA) was performed with parameters estimated using the least squares method with a polychoric correlation matrix. This method is used for ordinal items and has less strict normality criteria.

The goodness of fit of the model was examined in terms of the normalized Chi-square, defined as the ratio between the Chi-square value and the number of degrees of freedom (χ2/df), Goodness of Fit Index (GFI), Adjusted Goodness of Fit Index (AGFI), Comparative Fit Index (CFI), Root Mean-Square Residual (RMR), Root Mean Standard Error Standardized (SRMR) and Root Mean Standard Error of Approximation (RMSEA). A good overall fit was considered if the values of fit: X2/df ratio < 3 [26]; GFI, AGFI, CFI, values ≥ 0.90 and RMRS, RMSEA ≤ 0.06, and between 0.06 and 0.08 a reasonable fit [27, 28]. In addition, convergent validity was assessed with the Working Alliance Inventory Short scale, therapist version [11].

### Reliability

To evaluate the internal consistency of the instrument at a general level and for each of the factors, ordinal alpha with a value greater than or equal to 0.70 were used [29].

Temporal or test-retest stability was examined within 7–15 days using the intraclass correlation coefficient (ICC) in a sample of 100 nurses. CCI values ranged from 0 to 1. Values equal to or greater than 0.70 were considered adequate [22].

## Results

### Participant characteristics

Finally, a total of 213 nurses were included in the study. The mean age of participants was 37.5 years (SD 9.8) and 75.6% were female. Over half of the nurses (62.0%) reported being specialists in mental health nursing. Up to 48.8% reported working in the hospital setting. Table 1 shows the sociodemographic characteristics of the study population.

**Table 1:** Sociodemographic characteristics of the study population (n = 213)

	n	%
**Age (SD)**	37.5 (9.8)	
**Sex**		
Women	161	75.6
Men	52	24.4
**Mental Health Nursing Specialty**		
Yes	132	62.0
No	81	38.0
**Area in which the care activity is carried out**		
Community level	75	35.2
Hospital environment	104	48.8
Undefined	34	16.0
**Work shift**		
Morning	98	46.0
Morning and afternoon	55	25.8
Afternoon	18	8.5
Night	4	1.9
Rotating schedule	38	17.8
**Workday**		
Complete	200	93.9
Partial	13	6.1
**Type of contract**		
Permanent	114	53.5
Interim	74	34.7
Replacement	25	11.7
**Years working in mental health**	11.1 (8.8)	
**Years worked in the current position**	6.1 (7.1)	
**Higher level university education**		
PhD	12	5.6
MsC	75	35.2
Degree	126	59.1
**Other specific mental health training**		
Postgraduate	39	18.3
Masters	133	62.4
None	41	19.2

### Construct validity

#### Confirmatory factor analysis (CFA)

**Fig. 1:**
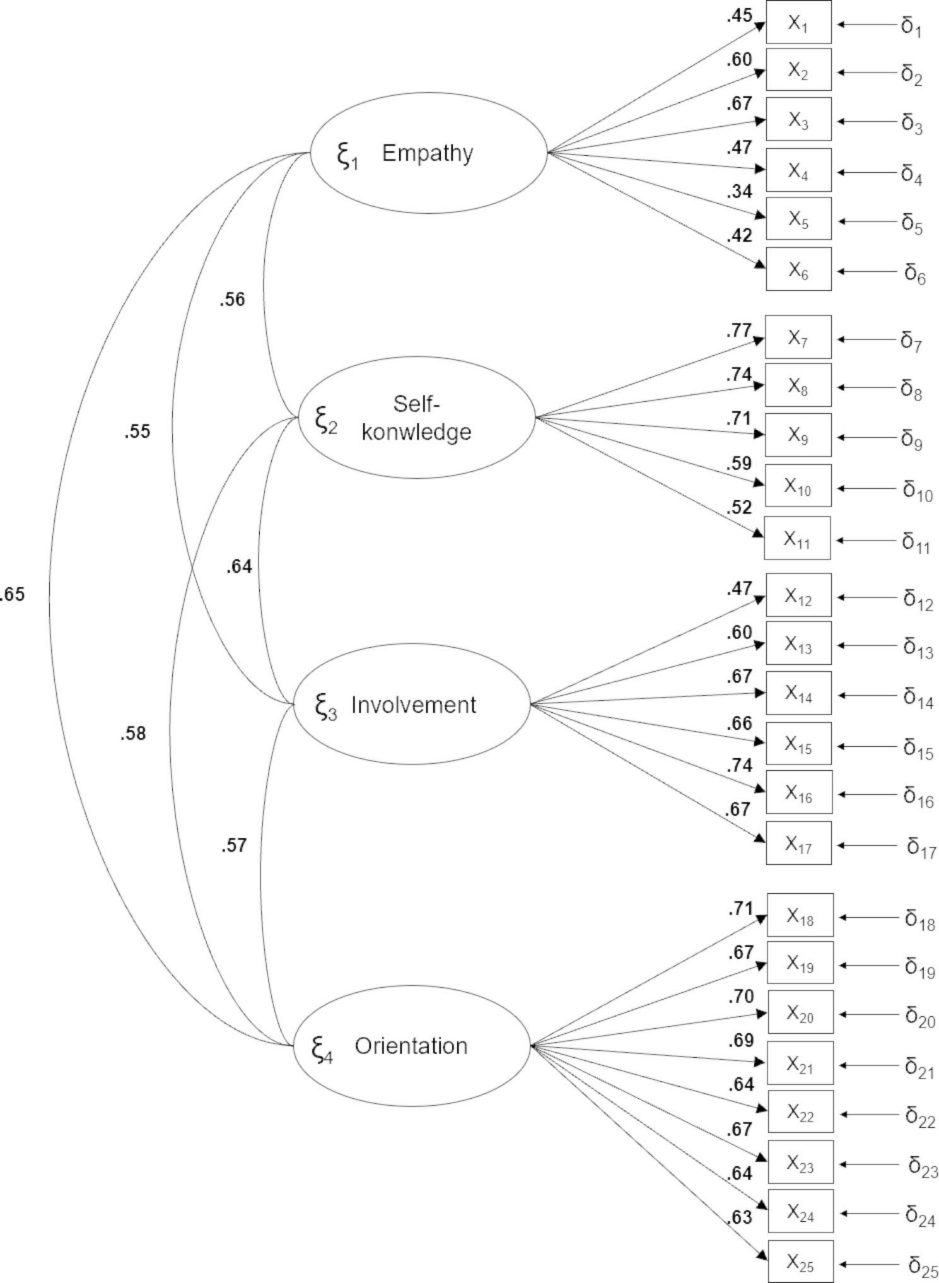
Factor loadings derived from the least squares estimation (least squares). Confirmatory factor analysis (λij)

In the confirmatory factor analysis, a four-factor model identical to the structure of the original version of the scale was proposed. Table 2 shows the model fit indices. Not all the indices showed an excellent fit, although they were acceptable. All item loadings were above 0.50, with the exception of items 1 (0.45), 4 (0.47), 5 (0.35), 6 (0.42) and 12 (0.47). Figure 1 shows the item loadings and the correlations between the factors of the TRAS-Nurse scale in Spanish.

**Table 2:** Goodness-of-fit indices for the confirmatory model Spanish QPC-OP

Index	Value
GFI	0.963
AGFI	0.955
CFI	0.771
RMR	0.035
SRMR	0.071
RMSEA	0.092
Ordinal’s alpha	0.939
Goodness of fit test	χ2 = 747.157; df = 269; p < .0001
Adjustment reason	χ2/df = 2.77

***GFI***: *Goodness-of-Fit Index.****AGFI***: *Adjusted Goodness-of-Fit Index.****CFI***: *Comparative Fit Index.****RMR***: *Root Mean-Square Residual.****SRMR***: *Standardized Root Mean-Square Residual.****RMSEA***: *Root Mean Square Error of Approximation.****df***: *Degrees of freedom.*

### Convergent validity

Spearman’s correlation coefficient between the two scales (TRAS-Nurse and WAI-S therapist version) was 0.670 (95% confidence interval: 0.584 to 0.742) p < .001, indicating good convergent validity.

### Reliability

Table 3 shows the values of ordinal alpha, and intraclass correlation coefficient. Regarding internal consistency, the ordinal alpha for the entire scale was 0.933, also reaching values above 0.70 in three of the four factors.

**Table 3:** Spanish TRAS-Nurse. Ordinal alpha, and intraclass correlation coefficient (n = 100)

Factor	Ordinal Alpha	ICC (95%CI)	95%CI
F1. Empathy	0.654	0.830	0.759–0.891
F2. Self-knowledge	0.798	0.860	0.793–0.906
F3. Involvement	0.801	0.919	0.879–0.945
F4. Orientation	0.866	0.944	0.917–0.962
Total	0.939	0.928	0.893–0.952

## Discussion

This study presents the results obtained in the analysis of the reliability and validity of the Spanish version of the TRAS-Nurse scale (Additional file 2). A translation and back-translation process was carried out to develop the adapted Spanish version of the TRAS-Nurse scale. The results of this phase were satisfactory and no difficulties were observed regarding the degree of comprehension or the administration of the instrument. Regarding psychometrics, the values of construct validity (CFA) were not quite good, but internal consistency and temporal stability (test-retest) were adequate.

The confirmatory factor analysis (CFA) carried out indicated that the Spanish version has the same four factors of the therapeutic relationship that were identified in the original Portuguese version of TRAS-Nurse [19]. However, the CFA showed some poor model fit indices. Regarding the convergent validity and considering that it can be considered adequate if a correlation with an instrument measuring the same construct is > 0.50 [30], we can conclude that there is convergent validity between the TRAS-Nurse and the WAI-S. When comparing the psychometrics of the Spanish versions of both assessment tools, it is clear that the WAI-S Therapist form (WAI-S-T) presents higher ordinal’s alpha coefficients for both overall measures and their corresponding scales (≥ 0.86) [31] compared to the TRAS-Nurse (≥ 0.62). Nonetheless, its overall ordinal’s alpha is higher than 0.90 which, according to some authors [32], may reflect unnecessary duplication of content across items and point more to redundancy than to homogeneity. Still, it should be noted that none of these assessment tools can replace the other, as the TRAS-Nurse focuses specifically on the nurse-patient relationship, whereas the WAI focuses on the relationship between the therapist, often a psychologist, and the patient.

Although we strived to conduct a methodologically robust study, several limitations should be noted. First, we used a convenience sample, which limited the representativeness of the sample and, consequently, the generalization of the results. Moreover, given the results obtained, more studies are needed to analyse the model fit of the instrument’s factor structure in the Spanish population.

## Conclusions

The TRAS-Nurse instrument was developed to highlight the importance of the relationship established between nurses and the people they care for, representing a tool that allows us to evaluate the quality of this relationship with rigor and systematization from the perspective of the nurse. This study found that the TRAS-Nurse, from the perspective of its psychometric properties, this tool is better suited to a four-factor model, as in the Portuguese version. Above all, it may identify which aspects require improvement in order to strengthen the therapeutic relationship and, consequently, improve the nursing care provided. Particularly in the context of Mental Health Nursing, in which the quality of the established therapeutic relationship is highly predictive of the success of nursing interventions, this seems to be an appropriate tool to be used in clinical contexts.

## Electronic supplementary material

Below is the link to the electronic supplementary material.

Additional file 1

Additional file 2

## Data Availability

The datasets used and/or analysed during the current study are available from the corresponding author on reasonable request.
