# Supplementary material for: Transcultural adaptation and psychometric properties of the spanish version of the therapeutic relationship Assessment Scale-Nurse
Source: BMC Nurs. 2023 Jul 28;22:248. doi: 10.1186/s12912-023-01412-8 (PMC10375615; doi:10.1186/s12912-023-01412-8)
Supplement: Supplementary file 1 — Additional file 1 [file 12912_2023_1412_MOESM1_ESM.docx]

| **Semantic equivalence of items from Portuguese to Spanish that were metrically validated** | | |
| --- | --- | --- |
| Item | Portuguese | Spanish |
| Item [1] | Apresento-me à/ao utente [I introduce myself to the patient] | Me presento a la persona que cuido |
| Item [2] | Pergunto à/ao utente como prefere ser tratada/o [I ask the patient what I should call him/her] | Pregunto a la persona que cuido cómo quiere ser tratada. |
| Item [3] | Esclareço a/o utente sobre o seu papel e o da/o enfermeira/o na relação [I inform the patient about his/ her role and that of the nurse in the relationship] | Clarifico a la persona que cuido su papel y el mío como enfermera en la relación. |
| Item [4] | Encorajo a/o utente a falar abertamente [I encourage the patient to speak openly] | Animo a la persona que cuido a hablar abiertamente. |
| Item [5] | Ajo de forma a alcançar a confiança da/o utente [I act in such a way that I gain the trust of the patient] | Intento que la persona que cuido confíe en mí. |
| Item [6] | Aplico os princípios éticos e deontológicos inerentes a uma relação terapêutica [I apply the ethical and deontological principles inherent to a therapeutic relationship] | Aplico los principio éticos y deontológicos propios de una relación terapéutica. |
| Item [7] | Compreendo e aceito a/o utente,  independentemente das suas  verbalizações [I understand and accept the patient, regardless of his/ her verbalizations] | Comprendo y acepto a la persona que cuido independientemente de aquello que verbaliza. |
| Item [8] | Apoio a/o utente, de igual forma, independentemente das suas verbalizações atuais e/ou passadas [I support the patient in the same way, regardless of his/her current and/or past verbalizations] | Apoyo a la persona que cuido independientemente de sus verbalizaciones actuales y/o pasadas. |
| Item [9] | Compreendo e aceito a/o utente, independentemente dos seus comportamentos [I understand and accept the patient, regardless of his/ her behaviour] | Comprendo y acepto a la persona que cuido al margen de sus comportamientos. |
| Item [10] | Apoio a/o utente, de igual forma, independientemente dos seus comportamentos atuais e/ou passados [I support the patient in the same way, regardless of his/her current and/or past behaviours] | Apoyo a la persona que cuido independientemente de sus comportamientos actuales y/o pasados. |
| Item [11] | Consigo compreender os sentimentos da/o utente [I can understand the patient's feelings] | Consigo comprender los sentimientos de la persona que cuido. |
| Item [12] | Evito a interferência dos meus problemas na relação com a/o utente [I do not let my problems interfere with the relationship with the patient] | Evito que mis problemas interfieran en la relación con la persona que cuido. |
| Item [13] | Aceito os sentimentos que experiencio na relação com a/o utente [I accept the feelings I experience in the relationship with the patient] | Acepto los sentimientos que experimento durante la relación con la persona que cuido. |
| Item [14] | Reconheço os meus pensamentos, sentimentos e comportamentos [I recognize my thoughts, feelings, and behaviours] | Reconozco los pensamientos, sentimientos y comportamientos que experimento en la relación con la persona que cuido. |
| Item [15] | Reflito sobre o potencial impacto dos meus pensamentos, sentimentos e comportamentos na relação com a/o utente [I reflect on the potential impact of my thoughts, feelings, na behaviours on the relationship with the patient] | Reflexiono sobre el posible impacto de mis pensamientos, sentimientos y comportamientos en la relación con la persona que cuido. |
| Item [16] | Reflito e identifico as minhas competências relacionais [I reflect on and identify my relational skills] | Reflexiono e identifico mis competencias relacionales. |
| Item [17] | Reflito e identifico as minhas limitações relacionais [I reflect on and identify my relational limitations] | Reflexiono e identifico mis limitaciones relacionales. |
| Item [18] | Garanto a identificação, junto da/o utente, das suas necessidades, expetativas e potencialidades [I guarantee the identification, along with the patient, of his/her needs, expectations, and potential] | Garantizo, junto a la persona que cuido, la identificación de sus necesidades, expectativas y potencialidades. |
| Item [19] | Ajudo a/o utente na identificação do seu problema [I help the patient to identify his/her problema] | Ayudo a la persona que cuido a identificar sus problemas. |
| Item [20] | Ajudo a/o utente na identificação de estratégias para lidar com / resolver o problema [I help the patient to identify strategies to deal with/ solve the problem] | Ayudo a la persona que cuido a identificar estrategias para mejorar o resolver su problema. |
| Item [21] | Ajudo a/o utente a identificar os fatores que estão na base da sua incapacidade para resolver o problema [I help the patient identify the factors that are at the base of his/her inability to solve the problema] | Ayudo a la persona que cuido a identificar los factores que pueden ser la base de su incapacidad para resolver su problema. |
| Item  [22] | Negoceio os objetivos a atingir com a/o utente [I negotiate with the patient the goals to be reached] | Negocio conjuntamente con la persona que cuido los objetivos a alcanzar. |
| Item [23] | Negoceio com a/o utente os contornos da intervenção [I negotiate with the patient the contours of the intervention] | Negocio conjuntamente con la persona que cuido los ámbitos de intervención. |
| Item [24] | Dedico ao utente o tempo que ela/e necesita [I dedicate to the patient the time he/she needs] | Dedico a la persona que cuido el tiempo que necesita. |
| Item [25] | Dedico ao utente a atenção que ela/e necesita [I dedicate to the patient the attention he/she needs] | Dedico a la persona que cuido la atención que necesita. |
