# Supplementary material for: Transcultural adaptation and psychometric properties of the spanish version of the therapeutic relationship Assessment Scale-Nurse
Source: BMC Nurs. 2023 Jul 28;22:248. doi: 10.1186/s12912-023-01412-8 (PMC10375615; doi:10.1186/s12912-023-01412-8)
Supplement: Supplementary file 2 — Additional file 2 [file 12912_2023_1412_MOESM2_ESM.docx]

**Therapeutic Relationship Assessment Scale-Nurse (TRAS-Nurse) – Spanish Version**

|  | 1 | 2 | 3 | 4 | 5 |
| --- | --- | --- | --- | --- | --- |
| Me presento a la persona que cuido. |  |  |  |  |  |
| Pregunto a la persona que cuido cómo quiere ser tratada. |  |  |  |  |  |
| Animo a la persona que cuido a hablar abiertamente. |  |  |  |  |  |
| Intento que la persona que cuido confíe en mí. |  |  |  |  |  |
| Comprendo y acepto a la persona que cuido independientemente de aquello que verbaliza. |  |  |  |  |  |
| Apoyo a la persona que cuido independientemente de sus verbalizaciones actuales y/o pasadas. |  |  |  |  |  |
| Comprendo y acepto a la persona que cuido al margen de sus comportamientos. |  |  |  |  |  |
| Apoyo a la persona que cuido independientemente de sus comportamientos actuales y/o pasados. |  |  |  |  |  |
| Consigo comprender los sentimientos de la persona que cuido. |  |  |  |  |  |
| Evito que mis problemas interfieran en la relación con la persona que cuido. |  |  |  |  |  |
| Acepto los sentimientos que experimento durante la relación con la persona que cuido. |  |  |  |  |  |
| Reconozco los pensamientos, sentimientos y comportamientos que experimento en la relación con la persona que cuido. |  |  |  |  |  |
| Reflexiono sobre el posible impacto de mis pensamientos, sentimientos y comportamientos en la relación con la persona que cuido. |  |  |  |  |  |
| Reflexiono e identifico mis competencias relacionales. |  |  |  |  |  |
| Reflexiono e identifico mis limitaciones relacionales. |  |  |  |  |  |
| Garantizo, junto a la persona que cuido, la identificación de sus necesidades, expectativas y potencialidades. |  |  |  |  |  |
| Ayudo a la persona que cuido a identificar sus problemas. |  |  |  |  |  |
| Ayudo a la persona que cuido a identificar estrategias para mejorar o resolver su problema. |  |  |  |  |  |
| Ayudo a la persona que cuido a identificar los factores que pueden ser la base de su incapacidad para resolver su problema. |  |  |  |  |  |
| Negocio conjuntamente con la persona que cuido los objetivos a alcanzar. |  |  |  |  |  |
| Negocio conjuntamente con la persona que cuido los ámbitos de intervención. |  |  |  |  |  |
| Dedico a la persona que cuido el tiempo que necesita. |  |  |  |  |  |
| Dedico a la persona que cuido la atención que necesita. |  |  |  |  |  |

A continuación, encontrará afirmaciones sobre lo que puede sentir o pensar hacia su paciente. Para cada afirmación, seleccione la respuesta con la que más se identifique: 1 - Nunca; 2 - Rara vez; 3 - A veces; 4 - A menudo; 5 - Siempre.
